# Development and Validation of Dietary Behavior Inventory—Surgery (DBI-S) in the Scope of International Post-Bariatric Surgery Guidelines and Recommendations

**DOI:** 10.3390/nu14183692

**Published:** 2022-09-07

**Authors:** Alexander Bäuerle, Laura Schräpler, Matthias Marsall, Gerrit Engelmann, Nadja Knoll-Pientka, Lynik Chantal Schüren, Marco Niedergethmann, Anita Robitzsch, Eva-Maria Skoda, Till Hasenberg, Martin Teufel

**Affiliations:** 1 Clinic for Psychosomatic Medicine and Psychotherapy, University of Duisburg-Essen, LVR-University Hospital Essen, 45147 Essen, Germany; 2 Center for Translational Neuro- and Behavioral Sciences (C-TNBS), University of Duisburg-Essen, 45147 Essen, Germany; 3 Institute for Patient Safety (IfPS), University Hospital Bonn, 53127 Bonn, Germany; 4 Department of Surgery, Obesity and Metabolic Surgery Center, Alfried-Krupp Hospital Essen, 45131 Essen, Germany; 5 Helios Obesity Center West, Helios St. Elisabeth Hospital Oberhausen, Witten/Herdecke University, Helios University Hospital Wuppertal, 42283 Wuppertal, Germany

**Keywords:** bariatric surgery, dietary behavior, validation, body mass index, cluster analysis, dietary recommendation, weight

## Abstract

(1) Dietary behavior is highly relevant for patients after bariatric surgery. No instrument exists assessing adherence to medical guidelines concerning the dietary behavior of patients after bariatric surgery. The aim of this study was to develop and validate such an instrument. (2) Data from patients after bariatric surgery (*n* = 543) were collected from March to May 2022. The development of the DBI-S was theory-based and interdisciplinary. Items’ and content validity of the DBI-S were examined. (3) The final version of the DBI-S consists of 13 items. Convergent validation was confirmed by significant correlations between DBI-S score and attitude towards healthy food (r = 0.26, *p* = <0.001) and impulsivity (r = −0.26, *p* = <0.001). Criterion validity was confirmed by significant correlations between DBI-S score and pre-/post-surgery BMI difference (r = −0.14, *p* = 0.002), pre-/post-surgery weight difference (r = 0.13, *p* = 0.003), and quality of life (r = 0.19, *p* = <0.001). Cluster analysis confirmed the ability to distinguish between two dietary behavior clusters (rather healthy and rather unhealthy). (4) The DBI-S is an economic and valid instrument to assess the adherence of post-bariatric surgery patients to the relevant dietary behavior recommendations and guidelines and can distinguish between rather unhealthy and healthy dietary behavior.

## 1. Introduction

Bariatric surgery is the most efficient therapy for generating long-term weight loss and decreasing mortality and comorbidity symptomatology in patients with grave obesity [1,2]. Therefore, the latest European and U.S. Guidelines for Obesity Management in adults recommended bariatric surgery according to BMI levels and associated obesity-related diseases [3,4]. As a result, the global number of patients receiving bariatric surgery has increased in the past years [5]. Studies show that the chosen procedure might also play a significant role in long-term weight loss outcome and that different procedures may lead to different outcomes at different times [6]. The most recent procedure is the Roux-en-Y gastric bypass, followed by sleeve gastrectomy and the one-anastomosis gastric bypass.

Nevertheless, a considerable amount of patients encounter long-term complications like limited weight loss outcome, weight regain over time, or negative psychological health consequences [7,8]. Empirical evidence suggests that, besides bariatric-surgery-induced changes in physiology, the long-term success of bariatric surgery strongly depends on the patients’ motivation to adhere to healthier dietary behavior [9,10,11,12]. As a consequence, the measurement of dietary behavior is an indicator for post-operative health outcome and long-term success of bariatric surgery and subsequent weight loss.

Dietary behavior can be described as a great variety of manners and attitudes towards nourishment and food intake [13]. It not only consists of the volume, nutritional value, and energy of the consumed food, but also of concrete and typical diet-related habits associated with dietary intake [13]. Engelmann and colleagues provided an overview of the different aspects regarding the assessment of dietary behavior in the general population and developed an instrument according to the guidelines of the World Health Organization [14].

Problematically, to date, there is no instrument available assessing adherence to relevant dietary behavior guidelines for post-bariatric-surgery patients. The application of the already validated “General Dietary Behavior Inventory” on this specific patient group is not possible, as the current dietary guidelines for bariatric surgery patients diverge strongly from those for a normal population group [13,15,16,17,18,19,20]. However, the assessment of dietary behavior after bariatric surgery is beneficial and important for bariatric surgery patients, as it might detect the indication of dietary and behavioral interventions, and thus prohibit the onset of problematic eating behaviors and negative long-term health outcomes [21]. As the long-term success of bariatric surgery strongly depends on the adherence to healthy dietary behavior [9,10,11,12], it is on behalf of both clinicians and patients to evaluate and initiate (healthy) dietary behavior after bariatric surgery in accordance with international guidelines and recommendations. A standardized dietary behavior inventory for patients after bariatric surgery should be applied as a standard in the scope of post-surgery medical examination in order to ensure appropriate post-surgical aftercare and long-term success of the treatment.

Therefore, the aim of this study was the development and validation of a standardised questionnaire, which addresses dietary behavior in patients after bariatric surgery. The Dietary Behavior Inventory—Surgery (DBI-S) for patients after bariatric surgery was developed based on nutritional recommendations for adult after bariatric surgery patients by Sherf-Dagan and colleagues, recently published in Advances in Nutrition [17], and on several medical guidelines published inter alia by the German Obesity Society (DAG) [15,16,20,22].

In their review, Sherf-Dagan and colleagues [17] gathered ongoing evidence and expert opinions on peri- and post-operative nutritional care to enhance the long-term success of bariatric surgery and prevent negative health outcomes. The given recommendations were divided into pre-surgery diet and supplementation, post-surgery diet, eating-related behaviors and gastrointestinal symptoms, and lifelong vitamin and mineral supplementation, as well as dietary recommendations. It became obvious that the long-term success of bariatric surgery strongly depends on the adherence to specific dietary behavior guidelines [17]. On this account, the DBI-S was designed under the conditions laid down in the given review by Sherf-Dagan and colleagues [17]. In order to follow the strong empirical evidence summarized by Sherf-Dagan and colleagues, the DBI-S was constructed based on their aggregated nutrition recommendations for patients after bariatric surgery.

To sum up, this study targets the following objectives. First, the goal of this study was to develop an instrument assessing dietary behavior in post-operative bariatric surgery patients, which evaluates the adherence to post-surgery dietary recommendations summarized by Sherf-Dagan and colleagues [17] and dietary behavior guidelines published inter alia by the DAG [15,16,17,20,22]. Second, it was the goal to examine convergent and criterion validity of the instrument. Third, the instrument should discriminate between healthy and unhealthy dietary behavior according to the mentioned recommendations and guidelines.

The developed DBI-S for the assessment of post-surgery dietary behavior is intended to represent the first instrument to assess the highly relevant adherence to the recommendations for adult bariatric surgery patients by Sherf-Dagan and colleagues [17] and several medical guidelines published inter alia by the DAG [15,16,20,22].

## 2. Materials and Methods

### 2.1. Development of the Dietary Behavior Inventory—Surgery

The DBI-S consists of a 19-item self-assessment questionnaire based on the above-mentioned recommendations and guidelines. For item generation, we summarised the recommendations and guidelines and compiled them into individual, distinguishable items. Subsequently, content validity and item relevance of the newly developed instrument were ensured in interdisciplinary expert panels, which consisted of two independent nutritionists, two physicians specialized in psychosomatic medicine, and two surgeons specialized in bariatric surgery, resulting in item exclusion and further adjustments of the initially generated items. Further psychological expertise in test construction was included. Aligned to the semantic differential [23], we implemented a five-point bipolar scale, in which participants had to select between concrete contrary dietary behaviors mirroring their average, daily dietary behavior. An example item is “I always eat in peace without being distracted by anything” compared with “While eating, I always distract myself with other things (for example watching TV, thoughts about work, reading“). See Tables S1 and S2 for the final version of the original items as well as the English translation of the items of the questionnaire. Each item was scored from 1 (=like behavior A) to 5 (=like behavior B). Several items were inverted to prevent assessment against response tendencies of participants and, therefore, improve data quality. Higher DBI-S scores represent greater adherence of patients after bariatric surgery to the relevant dietary behavior recommendations and guidelines.

Accordingly to the construction of the previously developed “General Dietary Behavior inventory” (GDBI and GDBI-E) [14,24], the DBI-S underlies a formative rather than a reflective measurement model. This is because each item of the DBI-S reflects a unique behavior resulting in a 19-item questionnaire covering very different behaviors, which may be not interrelated with each other [25].

### 2.2. Participants and Study Design

A cross-sectional study compromising an online survey was conducted from March to May 2022 via Unipark (Tivian XI GmbH). Participants were recruited at the Obesity and Metabolic Surgery Centre of Alfried Krupp Hospital, Essen, Germany and in exclusive topic-related social media groups. The following inclusion criteria were applied: undergone bariatric surgery, internet access, and age > 18 years. Patients who indicated they had not undergone bariatric surgery could not proceed with the assessment. Further, we only included data sets containing complete responses of all DBI-S items for the data analysis. Two participants with a pre-surgery BMI below 30 were excluded, as well as one participant who indicated a (obviously wrong) post-surgery weight of 9 KG. Moreover, we performed an outlier analysis for response time for the questionnaire and excluded 35 participants based on this to foster data quality. The final sample consisted of N = 543 participants. To complete the assessment took approximately 13.08 min on average (SD 4.06 min). The completion rate was 88%. Before starting the survey, electronic informed consent was obtained from all participants. The study was conducted according to the Declaration of Helsinki and approved by the Ethics Committee of the Medical Faculty of the University of Duisburg-Essen (20-9718-BO).

### 2.3. Study Variables

We used a series of items and scales to capture the convergent and criterion-related constructs regarding dietary behavior after bariatric surgery. Namely, convergent validity was tested measuring impulsivity on an eight-item scale (Cronbach’s alpha = 0.71) [26] and a single item capturing the attitude towards healthy food [27]. Further, to evaluate criterion validity, we gathered data on our participants’ body weight and body height to calculate their body mass index (BMI). In addition, a single item was deployed to assess the overall quality of life [28]. Several nutrition-related and socio-demographic variables for sample description purposes were assessed. All data are based on self-reports.

### 2.4. Statistical Analysis

R, RStudio, and several packages were used to perform the statistical procedures. In a first step, data pre-processing was performed, and the BMI was calculated based on the participants’ answers of body weight and body height. Participants were asked for their body weight prior to the bariatric surgery. The pre-/post-bariatric surgery BMI and body weight differences were calculated accordingly. In a second step, descriptive statistics and inferential statistics were conducted for sample description and item analysis. On this basis, the DBI-S score was computed as a sum score of all eligible DBI-S items after item analysis. Before the evaluation of interrelations of the DBI-S score with socio-demographic variables as well as construct and criterion validity variables, we examined whether any of the socio-demographic variables interfered the validation-relevant correlations. For this case, we computed partial correlation with the interfering variable considered as a covariate.

Because of the formative character of the DBI-S, common procedures to evaluate validity and reliability of an instrument as factor analysis or Cronbach’s alpha were not applicable [29]. Therefore, in a third step, a cluster analysis (k-means) was performed to evaluate the ability of the DBI-S to differentiate between adherent and non-adherent post-surgery dietary behavior. The number of clusters was set based on the elbow method [30]. Evaluation of the cluster assignment was performed using the silhouette coefficient [31]. Subsequently, cluster assignment was used as a nominal variable to re-evaluate the interrelations of the DBI-S clusters with the construct and criterion validity variables. All analyses considered a significance level *p* = 0.05, except those for which the alpha level was corrected for multiple testing. As other variables as the DBI-S items contained missing data, list-wise deletion was used for inferential statistics.

## 3. Results

### 3.1. Sample Characteristics

Participants of this study had a mean age of M = 46.8 years (SD = 10.0, Min = 22, Max = 72). The average pre- and post-surgery body weights were M = 147.5 kg (SD = 26.7) and M = 102.8 kg (SD = 25.2), respectively. The average pre-to-post-surgery weight loss of M = −44.7 kg (SD = 21.9) was significant (t_487_ = 45.08, *p* < 0.001). The average pre- and post-surgery BMIs were M = 51.2 kg/m^2^ (SD = 7.9) and M = 35.6 kg/m^2^ (SD = 7.8), respectively. Consistently, the pre-to-post-surgery difference in BMI (M = −15.6 kg/m^2^, SD = 7.5) was significant: t_487_ = 45.78, *p* < 0.001. Table 1 presents the characteristics of the study sample.

### 3.2. Descriptive Statistics of the DBI-S

Before analysis of the DBI-S items, we recoded the inverted items (nos. 15 and 18). The results of the item analysis are summarized in Table 2. We identified six items (nos. 2, 3, 8, 10, 13, and 15) that had insufficient characteristics, i.e., low variance and ceiling effects. Consequently, these items were excluded and the DBI-S score was calculated with the remaining 13 items.

The DBI-S score was calculated as the sum score over all remaining 13 items. The possible range of the DBI-S score is 13 to 65. The lowest/highest individual scores were 23 and 65 in the study sample, respectively.

### 3.3. Relationships between the DBI-S Score and Sociodemographic Variables

The DBI-S score was significantly related to participants’ age (r = 0.18, *p* < 0.001). Intercorrelations between the DBI-S score and other sociodemographic variables are presented in Table 3.

The results revealed that the DBI-S score was strongly related to the time since bariatric surgery. The post-hoc test (Hochberg’s GT2, which is recommended for unequal sample sizes between groups [32]) showed that the DBI-S score differed significantly between all groups, except for the group ‘Less than 12 months’ compared with the group ‘Less than 24 months’. Detailed results are presented in Supplementary Material Table S3.

### 3.4. Validity Test of the DBI-S Score

As the variables ‘age’ and ‘time since bariatric surgery’ were strongly related to the DBI-S score, we performed a preliminary analysis in which we tested whether these variables were related to any of the validation scales/variables. Age was significantly correlated with impulsivity (r = −0.10, *p* < 0.05). Time since bariatric surgery was significantly interrelated with all validation variables (see Table 4).

Consequently, subsequent analyses used age as a covariate variable regarding impulsivity, whereas time since bariatric surgery was used as a covariate variable regarding every validation variable. The results of the partial correlation analyses are shown in Table 5. As we conducted five individual analyses, we considered a Bonferroni-corrected significance level of *p* = 0.010 as significant in of these analyses. Additionally, Supplementary Material Figure S1 contains correlation plots to show the relationships between the DBI-S score and each validation variable by the four groups of time since bariatric surgery.

All correlations between DBI-S score and validation scales were significant in consideration of the corrected significance level.

### 3.5. Cluster Analysis and Validation Analysis of Cluster Assignment

**Figure 1:**
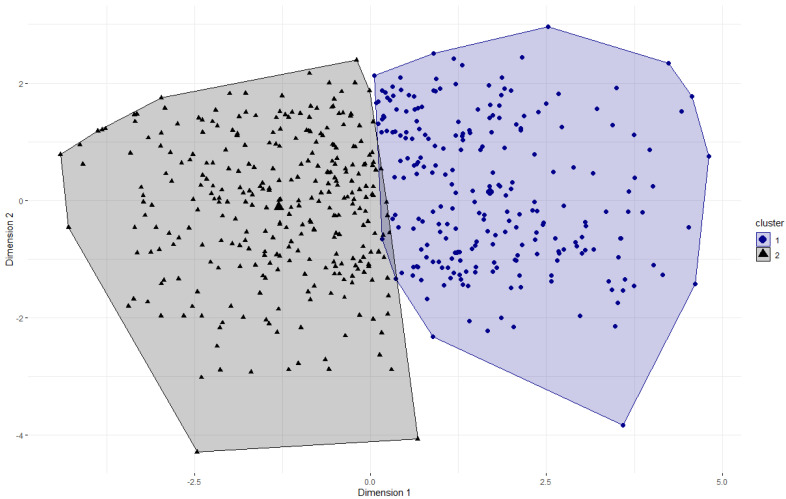
Partitioning clustering plot of the DBI-S with two clusters. A principal component analysis was used to determine two dimensions for visualization of the two-dimensional graph. The numbers on the X-/Y-axes indicate the z-scaled values of the dimensions. The cluster centers are displayed by larger symbols.

The elbow method indicated that two clusters would represent the 13 DBI-S items best. Therefore, we conducted the k-means clustering algorithm with two clusters, resulting in an average silhouette coefficient of M = 0.16. Figure 1 shows the partitioning clustering plot of the two clusters. Cluster 1 (*n* = 236) represents more non-adherent dietary behavior regarding the recommendations, whereas cluster 2 (*n* = 307) represents more adherent dietary behavior.

**Figure 2:**
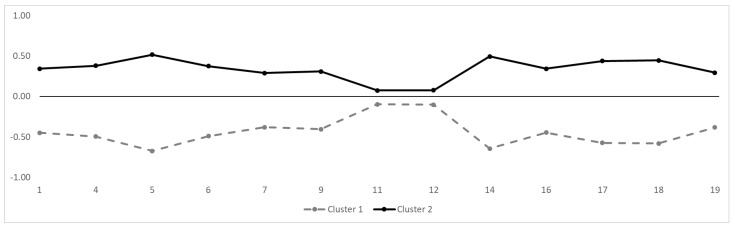
Cluster distribution of the 13 DBI-S items. The Y-axis indicates the z-standardized mean per item. The X-axis shows the item numbers.

Figure 2 shows the 13 items of the DBI-S on the two clusters on a z-standardized scale. Items 11 and 12 are able to discriminate between the two clusters because both items have a negative expression in cluster 1 and a positive expression in cluster 2. However, items 11 and 12 discriminate the least of all items.

Subsequently, we tested whether the cluster assignment was correlated with the validation scales. In accordance with the previous analyses regarding the DBI-S score, we used ‘age’ as well as ‘time since bariatric surgery’ as covariate variables within partial correlation analyses. Again, we corrected the significance level due to repeated analyses to *p* = 0.010. The results are presented in Table 6.

Considering the corrected significance level, ‘attitude towards healthy food’, ‘impulsivity’, ‘pre-/post-surgery BMI difference’, and ‘pre-/post-surgery body weight difference’ revealed significant relations with the DBI-S cluster assignment, whereas ‘quality of life’ did not (because of the applied Bonferroni-corrected significance level of *p* = 0.010).

## 4. Discussion

The goal of the study was to develop and validate the first instrument to measure adherence to relevant recommendations [17] and medical guidelines [15,16,20,22] for dietary behavior after bariatric surgery. To this end, the study aimed to confirm content, convergent, and criterion validity of the newly developed DBI-S. In addition, the study aimed to investigate whether the DBI-S can distinguish between rather unhealthy and healthy dietary behavior. To the best of our knowledge, this is the first study to pursue these goals in this context.

We were able to develop a theory-based instrument with confirmed content validity by strictly following the scientific recommendations for the development of such instruments [23,33]. Interdisciplinary experts reviewed the DBI-S in several review rounds. The economic DBI-S recognizes concrete and typical dietary behaviors and does not rely on the reconstruction of retrospective exceptional situations within the final 13 items. Furthermore, we were able to verify convergent as well as criterion validity by conducting several correlational analyses with measurements representing similar constructs and relevant health outcomes of dietary behavior. In addition, this study confirms the ability of the DBI-S to distinguish between rather unhealthy and healthy dietary behavior according to the named recommendations and guidelines in a sample of post-bariatric surgery patients.

The results implicate the important role of time since the bariatric surgery was conducted. Looking closely at the results of the performed analyses, the DBI-S cannot capture concrete dietary behavior in the group of participants who stated that the bariatric surgery took place less than six months ago. Considering the severe restrictions regarding nutritional aspects as well as dietary behavior shortly after a bariatric surgery, these results are valid in the context of clinical experience [15,16,17,20,22]. Restrictions concerning nutritional aspects include the consumption of only liquids and mashed or puréed foods in the first two weeks after surgery, transitioning to very small portion sizes distributed over four to six small meals a day, a higher than average protein intake, and the supplementation of different vitamins and minerals [3,16,17,18,20,22]. From the results, it can be inferred that there is a period of around six months needed to see significant changes in dietary behavior, impulsivity, BMI, body weight, and attitude towards healthy food, which may be explained by food intolerances within the first month after surgery. To sum up, these results strengthen the validity of the instrument.

Therefore, the analyses were controlled for time since the bariatric surgery. Supplementary Material Figure S1 illustrates these correlations and shows the relationships between the DBI-S score and each validation variable by the four groups of time since bariatric surgery. It is clearly shown that especially the weight loss success (represented by pre-/post-surgery body weight and pre-/post-surgery BMI difference) does not depend on the adherence to post-surgery nutrition recommendations for patients with less than six months passed since bariatric surgery (group 1). This pattern is completely different for the other groups (more than six months since bariatric surgery), concluding that the DBI-S is able to assess dietary behavior in patients who have had bariatric surgery at least six months ago, but not in patients in the first 6 months after bariatric surgery. This finding is valid from a clinical point of view considering the massive restrictions after bariatric surgery as well as likely food intolerances in this period [17].

Furthermore, the correlation plots shown in Supplementary Material Figure S1 indicate that, the more time elapsed since bariatric surgery, the more patients benefit from adherence to dietary guidelines and recommendations. These results can be underpinned by significant positive correlations between attitude towards healthy food and quality of life (only in regards to the DBI-S score-related validity test), and by significant negative correlations between impulsivity, pre-/post-surgery BMI difference, and pre-/post-surgery bodyweight difference, while these relations were controlled for time since bariatric surgery.

In consequence of the application of a very conservative Bonferroni-corrected significance level of *p* = 0.01, the outcome ‘quality of life’ did not show a significant relation with the DBI-S cluster assignment (Table 6). Considering the given *p*-value of 0.013, it is nevertheless plausible to presume a given tendency towards a significant positive relation between time passed since bariatric surgery and quality of life. However, looking at the correlations analysis to verify criterion validity, quality of life is strongly correlated with the DBI-S score.

Considering the non-significant difference in the DBI-S score, it can be concluded that the DBI-S is able to assess the dietary behavior independently of the surgery method. Therefore, future trials investigating weight loss after surgery could include the DBI-S as an additional co-variable.

Looking at the methodical framework of the DBI-S, a formative measurement model was applied rather than a reflective model. Because dietary behavior reflects many different aspects, which are incoherent, the formative measurement model was indicated. Following, it is not possible to assess a factorial structure of the DBI-S, which is not common in the development of psychometric instruments.

This study represents important strengths. The developed DBI-S reflects the most important aspects of dietary behavior in the context of post-bariatric-surgery patients. The instrument is based on theoretical reliable recommendations and clinical guidelines [15,16,20,22]. Therefore, the development process followed a deductive procedure on the relevant theoretical and clinical information. To the best of our knowledge, this is the first instrument measuring dietary behavior in the clinical vulnerable post-bariatric-surgery patient group by assessing more than just nutrient intake. Furthermore, the DBI-S is an economic tool to use in clinical practice and research projects. The instrument compromises 13 items and can be completed in under 5 min. The easy to evaluate and to interpret DBI-S score (sum-score) gives clinicians and researchers an economic and reliable information source regarding the adherence to respective recommendations and guidelines. Based on the results of the DBI-S, clinicians will be able to derive interventions for a more adherent diet. The interpretation of the results can be conducted independently of the bariatric surgery method used. As our results indicate that the DBI-S is a change-sensitive instrument, clinicians and researchers will be able to assess potential changes in dietary behavior over time, and thus evaluate general changes as well as the efficacy of dietary behavior interventions.

When interpreting the results of our study, limitations should be considered. The cross-sectional study design used in this study does not allow to consider causality. Thus, the interpretation of the results does not allow causal interpretation, but shows association between the constructs. Future studies should include the DBI-S and implement a longitudinal study design. In this case, the prognostic validity of the DBI-S could be assessed. Furthermore, the sensitivity regarding changes (e.g., due to a dietary behavior intervention) could be examined. Because of the online-based data collection, a possible selection bias cannot be ruled out. Therefore, the recruitment method might led to a selection of people who were familiar with the Internet use. However, the data collection was additionally performed at the Obesity and Metabolic Surgery Center of Alfried Krupp Hospital, Essen, Germany. Therefore, not only participants’ recruitment via online channels was performed. The online data collection was solely performed in topic-related online platforms. The gender distribution highly in favour of female participants is a relevant limitation of the results. This unequal gender distribution makes it impossible to examine a gender effect. However, the gender distribution represents the clinical reality. In fact, the gender distribution in this sample is comparable to the gender distribution in other samples of patients obtaining bariatric surgery [34]. All data were self-reported. Therefore, the possibility of biased reports should be considered. Looking at the DBI-S, methodological limitations arise. Because each item represents a specific aspect of dietary behavior, only complete DBI-S can be analyzed. Therefore, assessing the Cronbach’s alpha of the DBI-S, which represents the most common indicator for the internal consistency of a psychometric instrument, is not indicated. Unexpectedly, some of the items derived from the theory-based recommendations showed insufficient item characteristics and were excluded. Nevertheless, these items may be relevant in other sample compositions and should be considered in future studies.

## 5. Conclusions

Adherence to dietary behavior recommendations and guidelines is highly relevant for patients after bariatric surgery. To date, there are no instruments to assess the adherence or non-adherence to the respected recommendations and guidelines. The newly developed DBI-S is able to close this critical gap in clinical practice and research. Our study suggests that the developed DBI-S is an economic and valid instrument to assess the adherence of patients after bariatric surgery to the relevant dietary behavior recommendations and guidelines. The obtained results indicate good psychometric properties. The DBI-S is able to distinguish between rather unhealthy and healthy dietary behavior in this sample, irrespective of the surgical procedure. Considering obesity a chronic disease and that adherence to healthy, guideline-conforming dietary behavior seems to decrease as the post-operative time increases, it is even more important to detect potential risk patients. This could be done using the DBI-S as a screening tool in the future to ensure an appropriate aftercare for every patient. Consequently, the newly developed DBI-S meets all requirements to be used both in clinical and scientific work in the future.

## Figures and Tables

**Table 1:** Study sample description.

Characteristics	Participants		
	N	%	Missing N (%)
Sex			57 (10.5%)
Female	435	80.1%	
Male	51	9.4%	
Marital status			57 (10.5%)
Married	284	52.3%	
Living in a relationship	97	17.9%	
Single	82	15.1%	
Other	23	4.2%	
Educational degree			57 (10.5%)
No qualification	2	0.4%	
Secondary school diploma	139	25.6%	
University entrance qualification	70	12.9%	
Completed vocational training	171	31.5%	
University degree	104	19.2%	
Kind of bariatric surgery			0 (0%)
Sleeve gastrectomy	302	55.6%	
Roux-Y gastric bypass	140	25.8%	
Omega loop bypass	61	11.2%	
Gastric band	5	0.9%	
Single anastomosis duodenal-ileal bypass with sleeve (SADI/S)	6	1.1%	
Biliopancreatic division	1	0.2%	
Other technique	28	5.2%	
Time since bariatric surgery			0 (0%)
Less than 6 months	148	27.3%	
Less than 12 months	98	18.0%	
Less than 24 months	88	16.2%	
More than 24 months	209	38.5%	
General nutrition			55 (10.1%)
Omnivore diet	453	83.4%	
Vegetarian diet	16	2.9%	
Vegan diet	2	0.4%	
Other	17	3.1%	
Food intolerance			55 (10.1%)
Yes	89	16.4%	
No	399	73.5%	

**Table 2:** Descriptive statistics of the DBI-S items.

Item	Mean	SD	Median	Skew	Kurtosis	Response Distribution				
						1	2	3	4	5
DBI-S1	2.85	1.22	3	0.04	−0.51	21%	6%	53%	6%	14%
DBI-S2 *	4.07	1.14	5	−0.98	0.15	5%	1%	29%	12%	53%
DBI-S3 *	4.17	1.05	5	−0.98	0.12	2%	3%	25%	15%	55%
DBI-S4	3.69	1.19	3	−0.43	−0.56	6%	3%	42%	12%	36%
DBI-S5	3.77	1.18	4	−0.54	−0.53	6%	5%	35%	15%	39%
DBI-S6	3.67	1.48	4	−0.65	−0.97	15%	4%	26%	7%	47%
DBI-S7	3.66	1.55	4	−0.68	−1.06	18%	4%	19%	9%	49%
DBI-S8 *	4.28	0.93	5	−0.92	−0.25	1%	1%	24%	16%	58%
DBI-S9	3.3	1.49	3	−0.26	−1.24	20%	6%	32%	8%	34%
DBI-S10 *	4.23	1.22	5	−1.42	0.88	7%	2%	19%	6%	66%
DBI-S11	4	1.02	4	−0.42	−0.93	1%	2%	37%	13%	46%
DBI-S12	3.66	1.17	3	−0.3	−0.73	5%	6%	43%	10%	36%
DBI-S13 *	4.21	0.98	5	−0.83	−0.34	1%	1%	29%	13%	56%
DBI-S14	2.78	1.2	3	0.07	−0.54	22%	9%	50%	8%	11%
DBI-S15 * †	4.54	1.01	5	−2.27	4.3	4%	2%	9%	6%	79%
DBI-S16	3.45	1.08	3	−0.02	−0.4	5%	5%	54%	10%	25%
DBI-S17	3.22	1.43	3	−0.23	−1.12	20%	5%	35%	12%	28%
DBI-S18 †	3.55	1.02	3	−0.32	0	5%	4%	44%	26%	21%
DBI-S19	3.8	0.96	4	−0.16	−0.73	1%	3%	41%	24%	31%
DBI-S score (13 items)	45.39	8.01	46	−0.23	−0.32					

Notes. * excluded items; † inverted items.

**Table 3:** DBI-S score and relation to sociodemographic, medical and nutrition variables.

Variable	Groups	Mean	SD	Test	*p*
Sex	Female	45.6	7.9	t_484_ = −0.15	0.88
	Male	45.8	7.2		
Marital status	Married	45.9	7.9	F_3,482_ = 0.52	0.67
	Living in a relationship	45.5	6.7		
	Single	45.6	8.5		
	Other	43.8	8.8		
Educational degree	No qualification	46.5	26.2	F_4,481_ = 1.32	0.26
	Secondary school diploma	46.8	7.2		
	University entrance qualification	44.5	73		
	Completed vocational training	45.3	8.5		
	University degree	45.4	7.4		
Kind of bariatric surgery	Sleeve gastrectomy	45.6	7.9	F_6,536_ = 1.96	0.07
	Roux-Y gastric bypass	45.7	8.1		
	Omega loop bypass	46.1	8.1		
	Gastric band	38.2	8		
	Single anastomosis duodenal-ileal bypass with sleeve (SADI/S)	42.5	5.5		
	Biliopancreatic division	32	-		
	Other technique	42.7	8.3		
Time since bariatric surgery	Less than 6 months	50.5	5.9	F_3,539_ = 44.56	<0.001
	Less than 12 months	46.5	6.9		
	Less than 24 months	44.2	6.9		
	More than 24 months	41.7	8.2		
General nutrition	Omnivore diet	45.5	7.9	F_3,484_ = 0.88	0.45
	Vegetarian diet	48.5	6.1		
	Vegan diet	44.5	9.2		
	Other	46.6	8.7		
Food intolerance	Yes	45.7	8.1	t_486_ = 0.50	0.62
	No	45.2	6.9		

**Table 4:** Analyses of variances with time since bariatric surgery as a group variable.

Variable	Test	*p*
Attitude towards healthy food	F_3,482_ = 3.1	<0.05
Impulsivity	F_3,482_ = 4.6	<0.01
Pre-/post-surgery BMI difference	F_3,482_ = 68.0	<0.001
Pre-/post-surgery body weight difference	F_3,482_ = 64.4	<0.001
Quality of life	F_3,482_ = 9.6	<0.001

**Table 5:** Partial Pearson correlation analyses of the DBI-S score.

Validity Scale	Covariates	Correlation Coefficient r	*p*
Convergent validity			
Attitude towards healthy food	time since bariatric surgery	0.36	<0.001
Impulsivity	age; time since bariatric surgery	−0.38	<0.001
Criterion validity			
Pre-/post-surgery BMI difference	time since bariatric surgery	−0.14	0.002
Pre-/post-surgery body weight difference	time since bariatric surgery	−0.13	0.003
Quality of life	time since bariatric surgery	0.19	<0.001

**Table 6:** Partial Pearson correlation analyses of the DBI-S clusters.

Validity Scale	Covariates	Correlation Coefficient r	*p*
Convergent validity			
Attitude towards healthy food	time since bariatric surgery	0.26	<0.001
Impulsivity	age; time since bariatric surgery	0.26	<0.001
Criterion validity			
Pre-/post-surgery BMI difference	time since bariatric surgery	0.13	0.005
Pre-/post-surgery body weight difference	time since bariatric surgery	0.12	0.009
Quality of life	time since bariatric surgery	0.11	0.013

## Data Availability

The data presented in this study are available on reasonable request from the corresponding author.

## Supplementary Materials

The following supporting information can be downloaded at https://www.mdpi.com/article/10.3390/nu14183692/s1, Table S1: Items and Scoring of the Dietary Behavior Inventory—Surgery (DBI-S); Table S2: Items and Scoring of the Dietary Behavior Inventory—Surgery (DBI-S) English items; Table S3: Results of the post hoc test (Hochberg’s GT2) regarding the relation between the DBI-S score and time since bariatric surgery; Figure S1: (a–e): Correlation plots to visualize relationships between the DBI-S score and each validation variable by the four groups of time since bariatric surgery.

## List of Abbreviations

DBI-S	Dietary Behavior Inventory—Surgery
DGE	German Nutrition Society
GDBI	General Dietary Behavior Inventory
GDBI-E	General Dietary Behavior Inventory (English version)
WHO	Word Health Organization
