# Supplementary material for: Development and Validation of Dietary Behavior Inventory—Surgery (DBI-S) in the Scope of International Post-Bariatric Surgery Guidelines and Recommendations
Source: Nutrients. 2022 Sep 7;14(18):3692. doi: 10.3390/nu14183692 (PMC9504912; doi:10.3390/nu14183692)
Supplement: Supplementary file 1 [file nutrients-14-03692-s001.zip › nutrients-1887083-supplementary.pdf]

Table S1. *Items and Scoring of the Dietary Behaviour Inventory-Surgery (DBI-S)*

Item Code	*Behavior A*	*Scoring*					*Behavior B*
DBI-S1	*Ich esse immer in Ruhe, ohne dass ich von irgendetwas abgelenkt werde.*	*5*	*4*	*3*	*2*	*1*	*Während des Essens beschäftige ich mich immer mit anderen Themen (z.B. Fernsehen, Gedanken an die Arbeit, Lesen).*
*DBI-S2**	*Ich nehme über den ganzen Tag verteilt 3-4 kleine Mahlzeiten mit ausreichenden Essenspausen zu mir.*	*5*	*4*	*3*	*2*	*1*	*Ich nehme über den Tag verteilt weniger, dafür aber größere Mahlzeiten zu mir.*
*DBI-S3**	*Beim ersten Anzeichen einer Sättigung höre ich auf zu Essen.*	*5*	*4*	*3*	*2*	*1*	*Ich esse weiter, auch wenn ich eigentlich schon satt bin.*
DBI-S4	*Bei der Einnahme meiner Mahlzeiten lasse ich mir immer Zeit.*	*5*	*4*	*3*	*2*	*1*	*Meine Mahlzeiten nehme ich immer schnell ein.*
DBI-S5	*Beim Essen nehme ich kleine Bissen, die ich lange durchkaue.*	*5*	*4*	*3*	*2*	*1*	*Ich nehme beim Essen große Bissen, die ich nur kurz kaue.*
DBI-S6	*Ich halte immer Getränkepause vor den Mahlzeiten (15 Minuten) und nach den Mahlzeiten (30 Minuten) ein.*	*5*	*4*	*3*	*2*	*1*	*Ich nehme während der Mahlzeiten Getränke zu mir.*
DBI-S7	*Ich nehme täglich mindestens 1,5 Liter Flüssigkeit (ausgenommen alkoholische Getränke bzw. zucker-/süßungsmittelhaltige/ kohlensäurehaltige Getränke) zu mir.*	*5*	*4*	*3*	*2*	*1*	*Ich nehme täglich deutlich weniger als 1,5 Liter Flüssigkeit (ausgenommen alkoholische Getränke bzw. zucker- /süßungsmittelhaltige/ kohlensäurehaltige Getränke) zu mir.*
*DBI-S8**	*Ich esse täglich eiweißreiche Lebensmittel (Soja-Produkte, Eier, Linsen, Hülsenfrüchte, Milchprodukte, Fleisch, Fisch).*	*5*	*4*	*3*	*2*	*1*	*Ich esse nie eiweißreiche Lebensmittel (Soja-Produkte, Eier, Linsen, Hülsenfrüchte, Milchprodukte, Fleisch, Fisch).*
DBI-S9	*Ich nehme täglich und in empfohlener Menge ein hochwertiges Eiweißpräparat zu mir.*	*5*	*4*	*3*	*2*	*1*	*Ich nehme nie Eiweißpräparate zu mir.*
*DBI-S10**	*Ich nehme täglich und in empfohlener Menge Multivitamin- und Mineralstoffpräparate zu mir.*	*5*	*4*	*3*	*2*	*1*	*Ich nehme nie Multivitamin- und Mineralstoffpräparate zu mir.*
DBI-S11	*Ich esse täglich Gemüse.*	*5*	*4*	*3*	*2*	*1*	*Ich esse nie Gemüse.*
DBI-S12	*Ich esse täglich Obst.*	*5*	*4*	*3*	*2*	*1*	*Ich esse nie Obst.*
*DBI-S13**	*Ich nutze immer pflanzliche Fette (z.B. Margarine und Öle auf Basis von Raps, Oliven, Sonnenblumen).*	*5*	*4*	*3*	*2*	*1*	*Ich nutze immer tierische Fette (Butter, Schmalz etc.).*
DBI-S14	*Ich esse nie Süßigkeiten (z.B. Schokolade, Kekse, Gebäck).*	*5*	*4*	*3*	*2*	*1*	*Ich esse täglich Süßigkeiten (z.B. Schokolade, Kekse, Gebäck).*

DBI-S15†*	*Ich trinke regelmäßig (täglich oder auch jedes Wochenende) Alkohol.*	*1*	*2*	*3*	*4*	*5*	*Ich trinke nie oder sehr selten (nur zu besonderen Anlässen) Alkohol.*
DBI-S16	*Ich esse grundsätzlich Vollkornprodukte (z.B. Vollkornnudeln, Vollkornbrot).*	*5*	*4*	*3*	*2*	*1*	*Ich esse grundsätzlich Weißmehlprodukte (z.B. „herkömmliche" Nudeln, Weißbrot/Graubrot).*
DBI-S17	*Ich trinke nie zucker-/ süßungsmittelhaltige Getränke (z.B. Säfte, Limonade, gesüßter Kaffee/Tee, Zero-Getränke).*	*5*	*4*	*3*	*2*	*1*	*Ich trinke täglich zucker-/ süßungsmittelhaltige Getränke (z.B. Säfte, Limonade, gesüßter Kaffee/Tee, Zero-Getränke).*
DBI-S18†	*Ich esse täglich Lebensmittel mit vielen Kalorien, wie z.B. Smoothies, Eiscreme, Milchshakes, Säfte, Schokolade, Kuchen, Kekse.*	*1*	*2*	*3*	*4*	*5*	*Ich esse nie Lebensmittel mit vielen Kalorien, wie z.B. Smoothies, Eiscreme, Milchshakes, Säfte, Schokolade, Kuchen, Kekse.*
DBI-S19	*Ich esse nie Fast-Food- und Fertigprodukte (z.B. Tiefkühlpizza, Mikrowellenfertiggerichte).*	*5*	*4*	*3*	*2*	*1*	*Ich esse täglich Fast-Food- und Fertigprodukte (z.B. Tiefkühlpizza, Mikrowellenfertiggerichte).*

** Items excluded for calculation of the DBI-S Score.*

† Inverted items

Table S2. Items and Scoring of the Dietary Behaviour Inventory-Surgery (DBI-S) English items

Item Code	*Behavior A*	*Scoring*					*Behavior B*
DBI-S1	*I always eat peacefully, without getting distracted.*	*5*	*4*	*3*	*2*	*1*	*While eating, I always distract myself with other things (e.g. watching TV, thinking about work, reading).*
*DBI-S2**	*Throughout the day, I eat 3-4 small meals with longer breaks in between.*	*5*	*4*	*3*	*2*	*1*	*Throughout the day, I eat less frequently, but bigger meals.*
*DBI-S3**	*When I recognize satiety, I stop eating immediately.*	*5*	*4*	*3*	*2*	*1*	*I continue eating, even when I am fed up.*
DBI-S4	*While eating, I always take my time.*	*5*	*4*	*3*	*2*	*1*	*I am always in a rush while eating.*
DBI-S5	*While eating, I take small bites, which I am chewing carefully.*	*5*	*4*	*3*	*2*	*1*	*While eating, I take big bites, which I am chewing briefly.*
DBI-S6	*I don't drink any beverages before (15 minutes) or after (30 minutes) meals.*	*5*	*4*	*3*	*2*	*1*	*I drink during mealtimes.*
DBI-S7	*Throughout the day, I drink at least 1,5l (except alcoholic beverages, or beverages containing sugars/artificial sweeteners/soda).*	*5*	*4*	*3*	*2*	*1*	*Throughout the day, I drink significantly less than 1,5l (except alcoholic beverages, or beverages containing sugars/artificial sweeteners/soda).*
*DBI-S8**	*I eat protein-rich foods daily (soy, eggs, lentils, legumes, dairy, meat, fish).*	*5*	*4*	*3*	*2*	*1*	*I never eat protein-rich foods (soy, eggs, lentils, legumes, dairy, meat, fish).*
DBI-S9	*I take a high-quality protein supplement daily in the recommended dosage.*	*5*	*4*	*3*	*2*	*1*	*I never take protein supplements.*
*DBI-S10**	*I take vitamin and mineral supplements on a daily basis in the recommended dosage.*	*5*	*4*	*3*	*2*	*1*	*I never take vitamin or mineral supplements.*
DBI-S11	*I eat vegetables daily.*	*5*	*4*	*3*	*2*	*1*	*I never eat vegetables.*
DBI-S12	*I eat fruit daily.*	*5*	*4*	*3*	*2*	*1*	*I never eat fruit.*
*DBI-S13**	*I always use vegetable fats (e.g. margarine and oils based on rape, olives or sunflower).*	*5*	*4*	*3*	*2*	*1*	*I always use animal fats (butter, lard).*
DBI-S14	*I never eat sweets (e.g. chocolate, cookies, pastry).*	*5*	*4*	*3*	*2*	*1*	*I eat sweets daily (e.g. chocolate, cookies, pastry).*

DBI-S15†*	*I drink alcohol regularly (daily or every weekend).*	*1*	*2*	*3*	*4*	*5*	*I never or rarely (only at special occasions) drink alcohol.*
DBI-S16	*I eat whole-grain products on principle (e.g. bread, pasta).*	*5*	*4*	*3*	*2*	*1*	*I eat white flour products on principle (e.g. bread, pasta).*
DBI-S17	*I never drink beverages, which contain sugar or artificial sweeteners (e.g. soda, lemonade, sweetened coffee, diet drinks).*	*5*	*4*	*3*	*2*	*1*	*I drink beverages that contain sugar or artificial sweeteners on a daily basis (e.g. soda, lemonade, sweetened coffee, diet drinks).*
DBI-S18†	*I eat high calorie foods daily (e.g. smoothies, ice cream, milkshakes, juices, chocolate, cake, cookies).*	*1*	*2*	*3*	*4*	*5*	*I never eat high calorie foods (e.g. smoothies, ice cream, milkshakes, juices, chocolate, cake, cookies).*
DBI-S19	*I never eat fast food or instant meals (e.g. frozen pizza, microwave instant meals).*	*5*	*4*	*3*	*2*	*1*	*I eat fast food or instant meals daily (e.g. frozen pizza, microwave instant meals).*

** Items excluded for calculation of the DBI-S Score.*

† Inverted items

Note: This translation is only for publication purpose. No validation of the translated items was performed.

Table S3. *Results of the post hoc test (Hochberg's GT2) regarding the relation between the DBI-S Score and time since bariatric surgery*

Compared groups	*Mean difference*	*Standard Error*	*P value*
Less than 6 months to Less than 12 months	*3.98*	*0.94*	*< 0.001*
Less than 6 months to Less than 24 months	*6.30*	*0.97*	*< 0.001*
Less than 6 months to More than 24 months	*8.77*	*0.77*	*< 0.001*
Less than 12 months to Less than 24 months	*2.33*	*1.06*	*0.16*
Less than 12 months to More than 24 months	*4.79*	*0.88*	*< 0.001*
Less than 24 months to More than 24 months	*2.47*	*0.91*	*0.035*

Figure S1. Correlation plots to visualize relationships between the DBI-S Score and each validation variable by the four groups of time since bariatric surgery

Group definition:
1 = Less than 6 months
2 = Less than 12 months
3 = Less than 24 months
4 = More than 24 months

a.

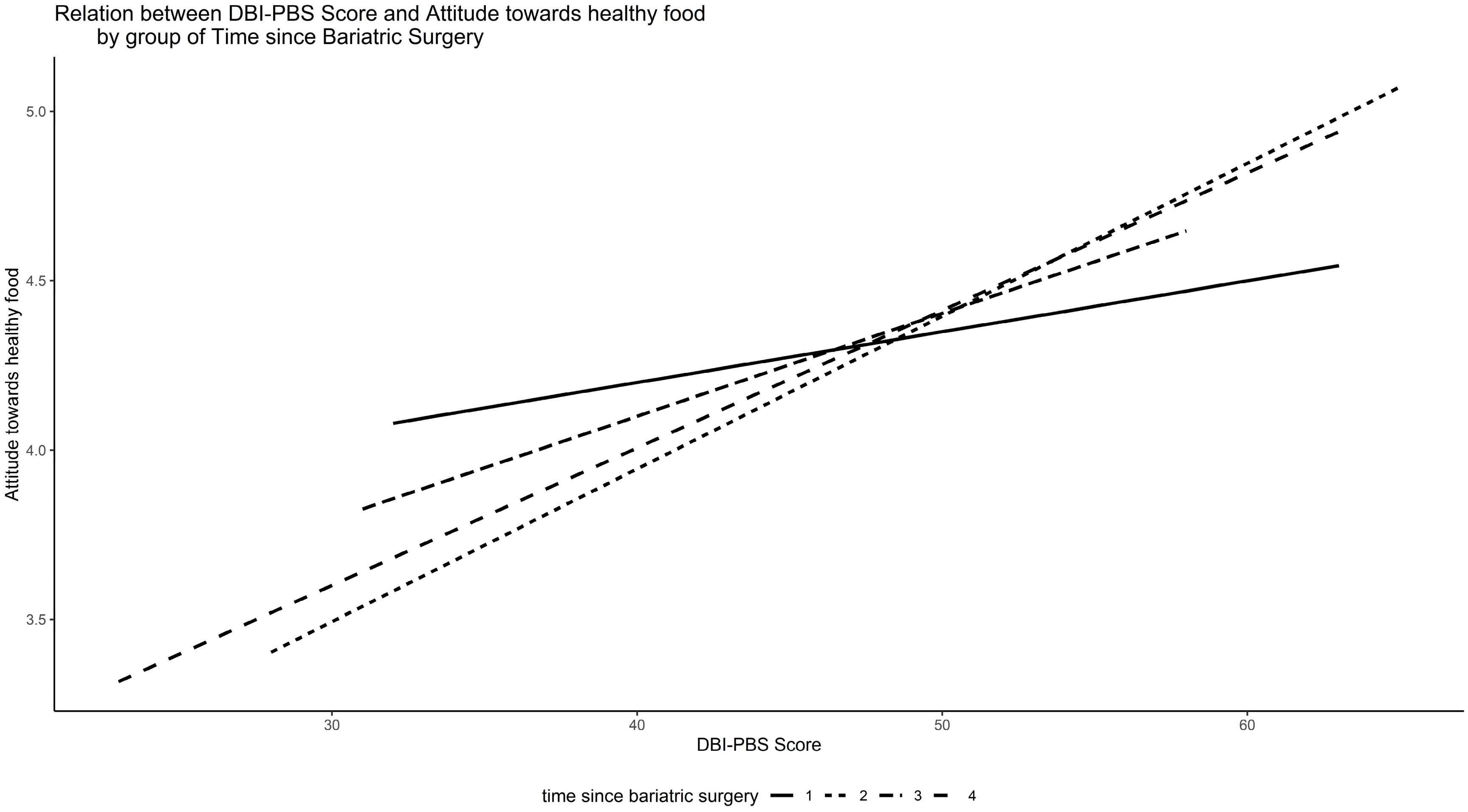

b.

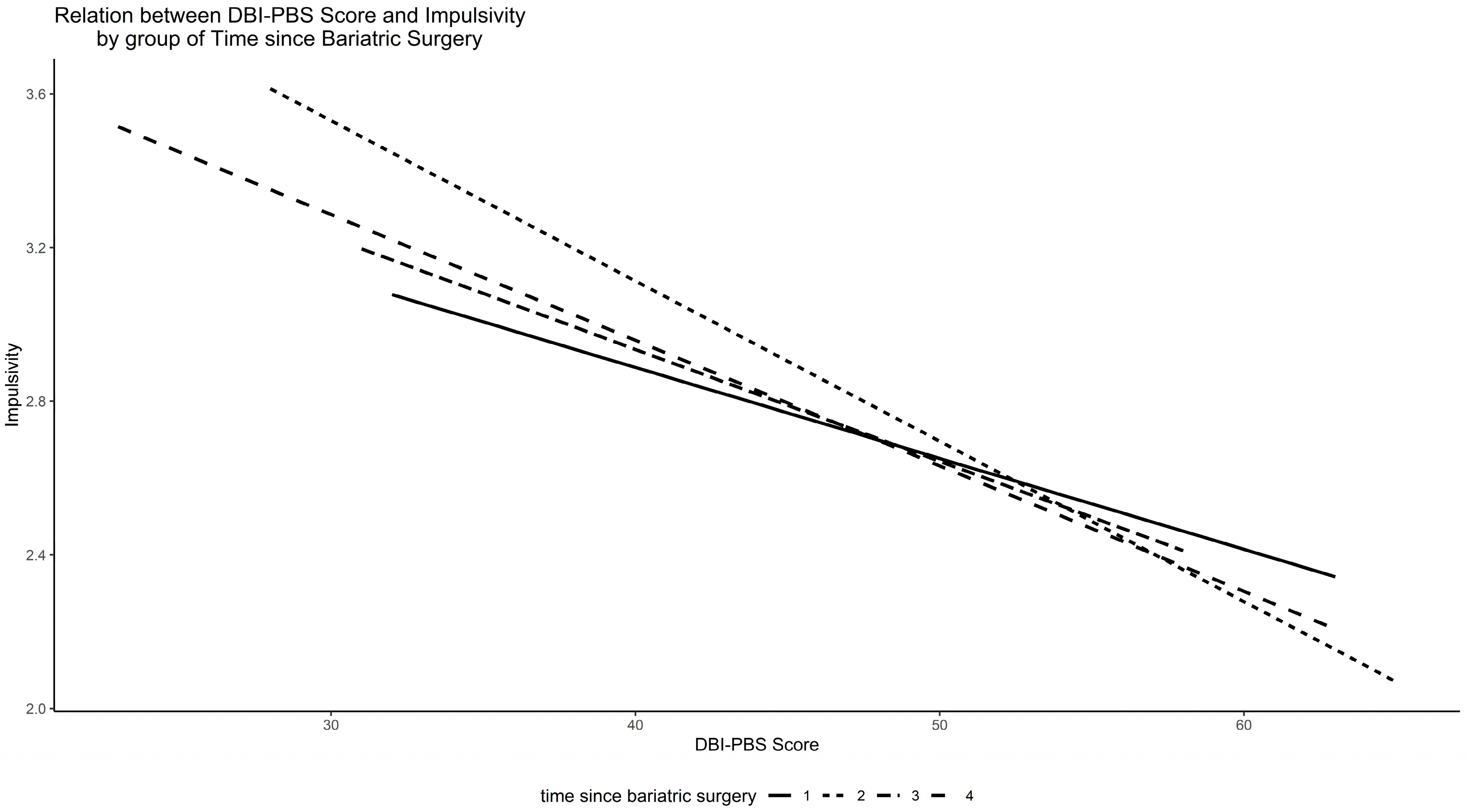
Relation between DBI-PBS Score and Impulsivity
by group of Time since Bariatric Surgery
Impulsivity
3.6
3.2
2.8
2.4
2.0
30
40
50
60
DBI-PBS Score
time since bariatric surgery
1
2
3
4

c.

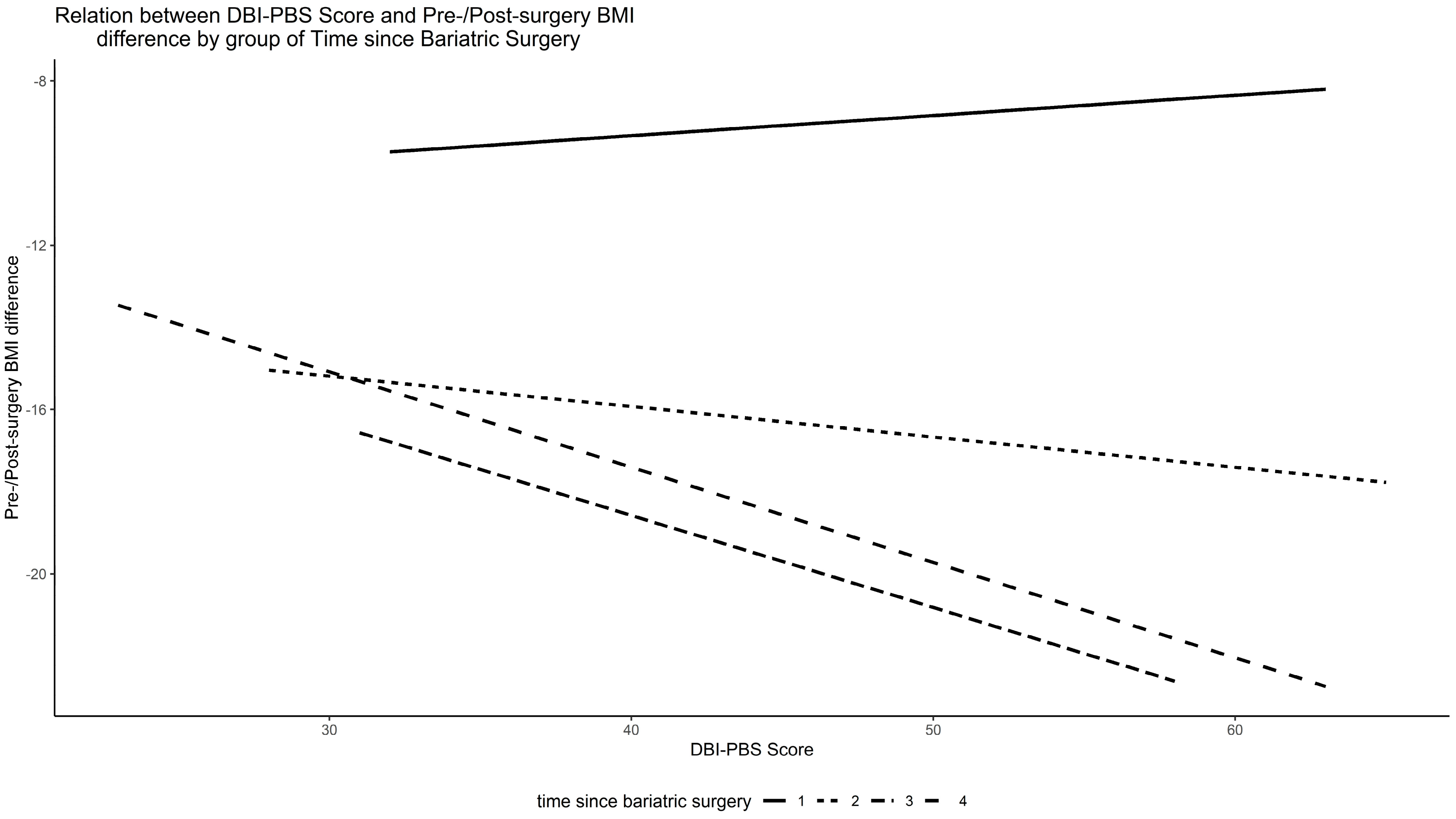
Relation between DBI-PBS Score and Pre-/Post-surgery BMI
difference by group of Time since Bariatric Surgery
-8
-12
-16
-20
Pre-/Post-surgery BMI difference
30
40
50
60
DBI-PBS Score
time since bariatric surgery
1
2
3
4

d.

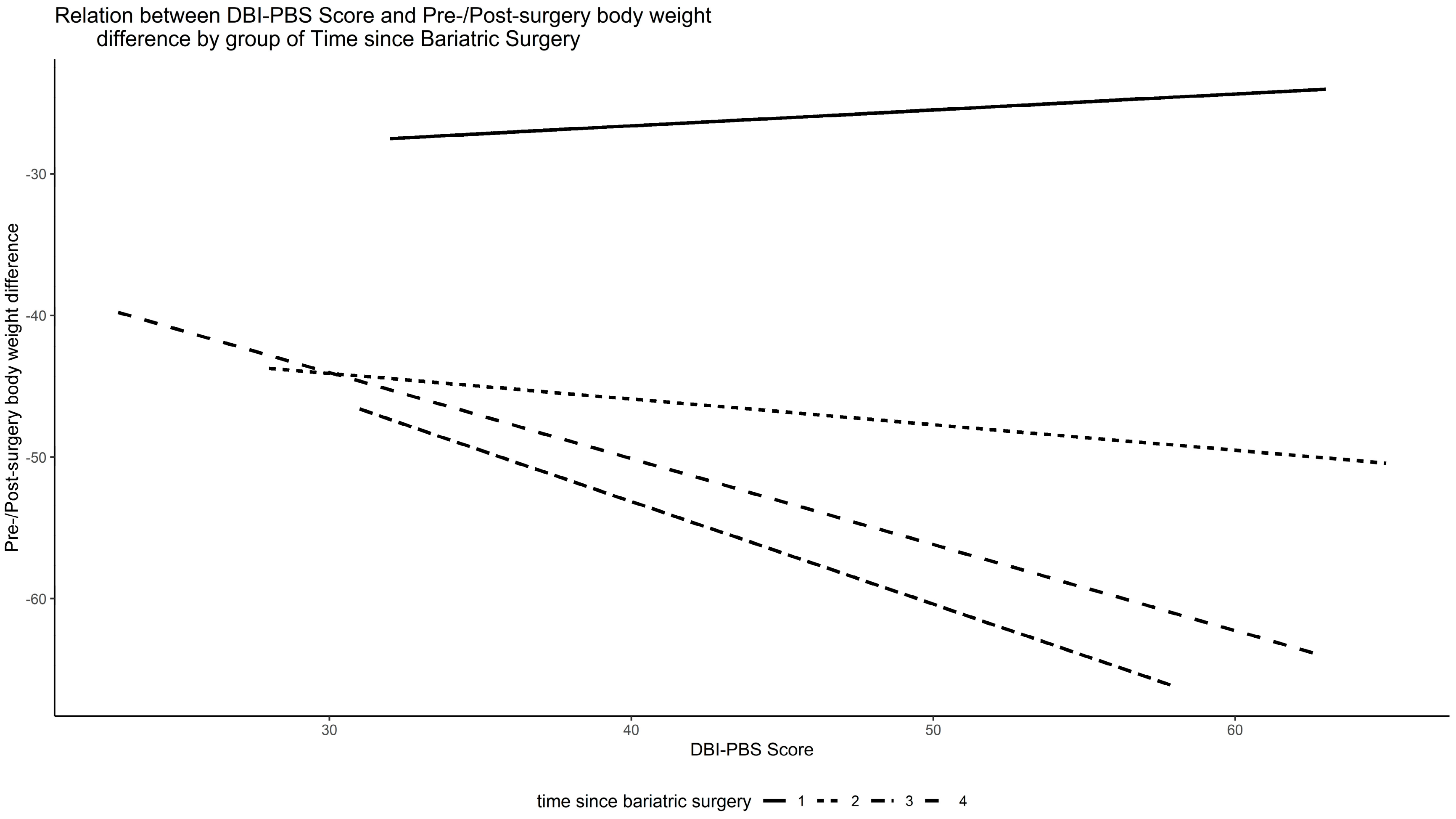
Relation between DBI-PBS Score and Pre-/Post-surgery body weight difference by group of Time since Bariatric Surgery
Pre-/Post-surgery body weight difference
-30
-40
-50
-60
30
40
50
60
DBI-PBS Score
time since bariatric surgery
1
2
3
4

e.

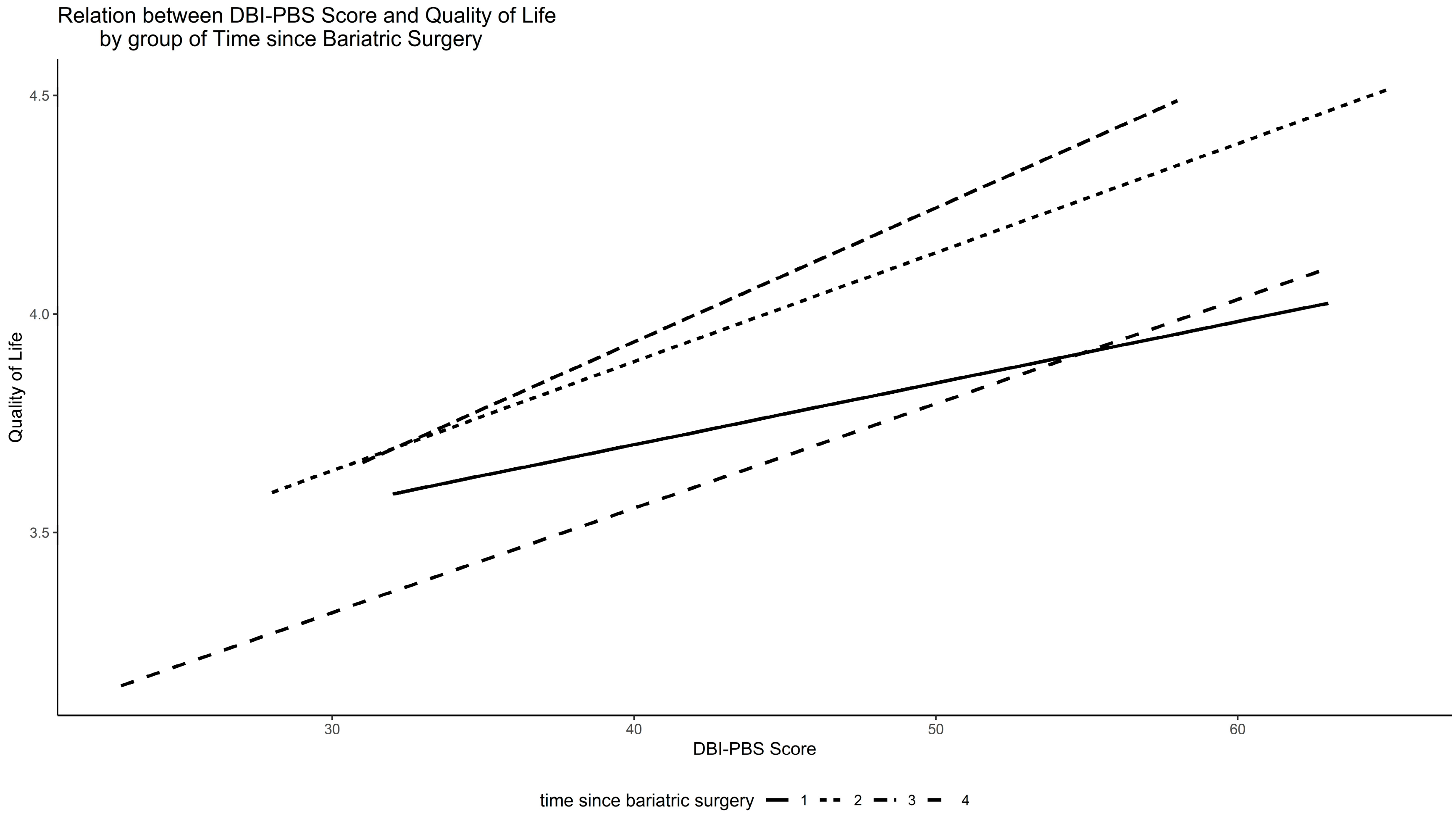

Note Figure S1a-e: DBI-PBS is equivalent to DBI-S
